# A New Model for Evaluation of Interventions to Prevent Obesity in Early Childhood

**DOI:** 10.3389/fendo.2019.00132

**Published:** 2019-03-01

**Authors:** Alison Hayes, Eng J. Tan, Thomas Lung, Vicki Brown, Marj Moodie, Louise Baur

**Affiliations:** ^1^Centre for Research Excellence in Early Prevention of Obesity in Childhood, Sydney, NSW, Australia; ^2^Faculty of Medicine and Health, The University of Sydney School of Public Health, Sydney, NSW, Australia; ^3^Health Economics and Process Evaluation, The George Institute for Global Health, University of New South Wales, Sydney, NSW, Australia; ^4^Deakin Health Economics, Centre for Population Health Research, School of Health and Social Development, Deakin University, Geelong, VIC, Australia; ^5^The Children's Hospital at Westmead Clinical School, University of Sydney, Sydney, NSW, Australia

**Keywords:** modeling, micro-simulation, obesity, epidemiology, economic evaluation

## Abstract

**Background:** Childhood obesity is a serious public health issue. In Australia, 1 in 4 children is already affected by overweight or obesity at the time of school entry. Governments around the world have recognized this problem through investment in the prevention of pediatric obesity, yet few interventions in early childhood have been subjected to economic evaluation. Information on cost-effectiveness is vital to decisions about program implementation. A challenge in evaluating preventive interventions in early childhood is to capture long-term costs and outcomes beyond the duration of an intervention, as the benefits of early obesity prevention will not be realized until some years into the future. However, decisions need to be made in the present, and modeling is one way to inform such decisions.

**Objective:** To describe the conceptual structure of a new health economic model (the Early Prevention of Obesity in CHildhood (EPOCH) model) for evaluating childhood obesity interventions; and to validate the epidemiologic predictions.

**Methods and Results:** We use an individual–level (micro-simulation) method to model BMI trajectories and the progression of obesity from early childhood to adolescence. The equations predicting individual BMI change underpinning our model were derived from data from the population-representative study, the Longitudinal Study of Australian Children (LSAC). Our approach is novel because it will account for costs and benefits accrued throughout childhood and adolescence. As a first step to validate the epidemiological predictions of the model, we used input data representing over 250,000 children aged 4/5 years, and simulated BMI and obesity trajectories until adolescence. Simulated mean BMI and obesity prevalence for boys and girls were verified by nationally-representative data on children at 14/15 years of age.

**Discussion:** The EPOCH model is epidemiologically sound in its prediction of both BMI trajectories and prevalence of obesity for boys and girls. Future developments of the model will include socio-economic position and will incorporate the impacts of obesity on healthcare costs. The EPOCH model will help answer: when is it best to intervene in childhood; what are the most cost-effective approaches and which population groups will benefit most from interventions.

## Introduction

Childhood obesity is a serious public health issue, with governments around the world beginning to invest in prevention programs. In Australia, similar to several other high-income countries, overweight and obesity affect approximately one in four children and adolescents (1). Due to the high prevalence of the problem globally (2), including in low and middle-income countries, the World Health Organization (WHO) established the Ending Childhood Obesity Commission. The Commission's report, published in 2016, put forward a comprehensive, integrated package of recommendations to address childhood obesity, largely through prevention interventions, both at levels close to the individual and family, and through more upstream approaches (3).

The WHO Commission emphasized the need for greater evidence in informing policy and actions targeted at reducing overweight and obesity in children (3). Since resources for prevention are limited, policy, and decision-makers also need evidence that interventions are cost-effective, i.e., that they represent value for money. Evidence gaps exist around what may be cost-effective in the 0–5 year age group, as very few preventive interventions in early childhood have been subject to economic evaluation (4–8). The reasons for this vary—including the difficulties in obtaining health care cost data, the paucity of health-related quality of life (HRQoL) instruments appropriate to this age group (6, 9) for use in cost-utility analysis, and the lack of validated models to project quality of life and BMI trajectories. The full benefits of obesity prevention in early childhood will not be fully realized until many years into the future, when chronic/obesity related disease manifests itself in adulthood, yet there is also evidence of shorter term impacts of childhood obesity on childhood cardiovascular risk factors (10, 11), insulin resistance in adolescence (12), and asthma (13). There is good evidence that rapid weight gain in early childhood tracks to later adolescent and adult obesity (14–17) so implementation of cost-effective interventions in early childhood could be key to slowing down the progression of obesity across the life course.

One of the challenges in evaluating interventions in early childhood is knowing how obesity progression may change in the longer term, as a result of the implementation of policies and interventions. Modeling approaches may contribute to evaluating both the effectiveness and the cost-effectiveness of obesity treatment and prevention programmes over a longer and more policy relevant timeframe than simply within the timeframe of a randomized controlled trial. Modeling is increasingly being used to guide policy decisions (18, 19) and is a very powerful tool to investigate the health and cost impacts of a range of interventions aimed at reducing childhood obesity (20). Models allow for the synthesis of evidence from different sources to simulate the effects of interventions under different scenarios and to predict both mid- and long-term outcomes.

As part of a program of work in the Center of Research Excellence in the Early Prevention of Obesity in Childhood (EPOCH–CRE), we are building a health economic model (EPOCH model) based on Australian data that can project child BMI trajectories and obesity, and estimate the future costs and cost savings that might be achieved from different interventions. Our model spans a time horizon extending from early childhood until late adolescence (4–15 years), thereby modeling the mid-term outcomes of obesity intervention in early childhood (i.e., the health benefits and healthcare cost-savings that we might expect to accrue up until late adolescence). The EPOCH model will therefore fill a gap in the literature on the cost-effectiveness of obesity intervention by including costs and benefits specific to the childhood and adolescent years, and will be complementary to modeling approaches that use a lifetime horizon to focus on the longer-term costs and benefits of prevention of obesity-related diseases. In the present paper, we describe the rationale and conceptual structure of the EPOCH model, which is an important part of the model building process (21). We also describe data sources that inform the model. Additionally, as a first step, we present simulations and internal validation of the core component of the model-the BMI trajectory model, which drives the epidemiologic progression of obesity.

## Conceptual Framework of the Epoch Model

A range of modeling methodologies have been used in economic evaluation of obesity prevention and treatment, yet there have been very few modeled economic evaluations for children (22). Most models take a Markov approach (22–24) in which populations are moved through the model as cohorts of identical population groups that transition through states representing healthy weight, overweight and obesity. A major contribution to this literature has been provided by the Assessing Cost Effectiveness (ACE) studies (25) which have used consistent methods and a common modeling platform (a proportional multi-state life table approach) to assess cost-effectiveness of a number of different interventions in childhood (26–28). These and other published models of childhood obesity use a lifetime time horizon (29), and mostly account for costs, health, and HRQoL consequences in adulthood.

Whilst the major health and economic impacts of childhood obesity are in later adulthood (30), there is ample evidence of more immediate impacts on health and health care costs during childhood (31–34). The EPOCH model accounts for these costs and uses a time horizon extending till late adolescence, thus projecting forward to a policy relevant time-frame. We will be able to compare interventions during early childhood and also beyond early childhood, for example with those targeted at primary school children or adolescents.

We have chosen an individual level (micro-simulation) approach which models members of a population separately and thus allows for population heterogeneity. This will enable us to model interventions that have different specific target groups (35), for example, teenage girls who are overweight and in lower socioeconomic groups, or interventions whose effect varies by individual characteristics. This is particularly important in obesity prevention and treatment, because of the strong socioeconomic patterning of obesity in Australia (36) and elsewhere.

The EPOCH model does not have the restriction of categorizing children into only three weight status groups, but accounts for the full distribution of body-mass index (BMI) in the modeled population at any point in time and the increasing right skew of this distribution over time. The emergence of severe obesity as a recent phenomenon among adolescents (36) means it is important to be able to predict the upper end of the BMI distribution, where health outcomes are generally poorer and costs are higher.

### EPOCH Model Structure

**Figure 1:**
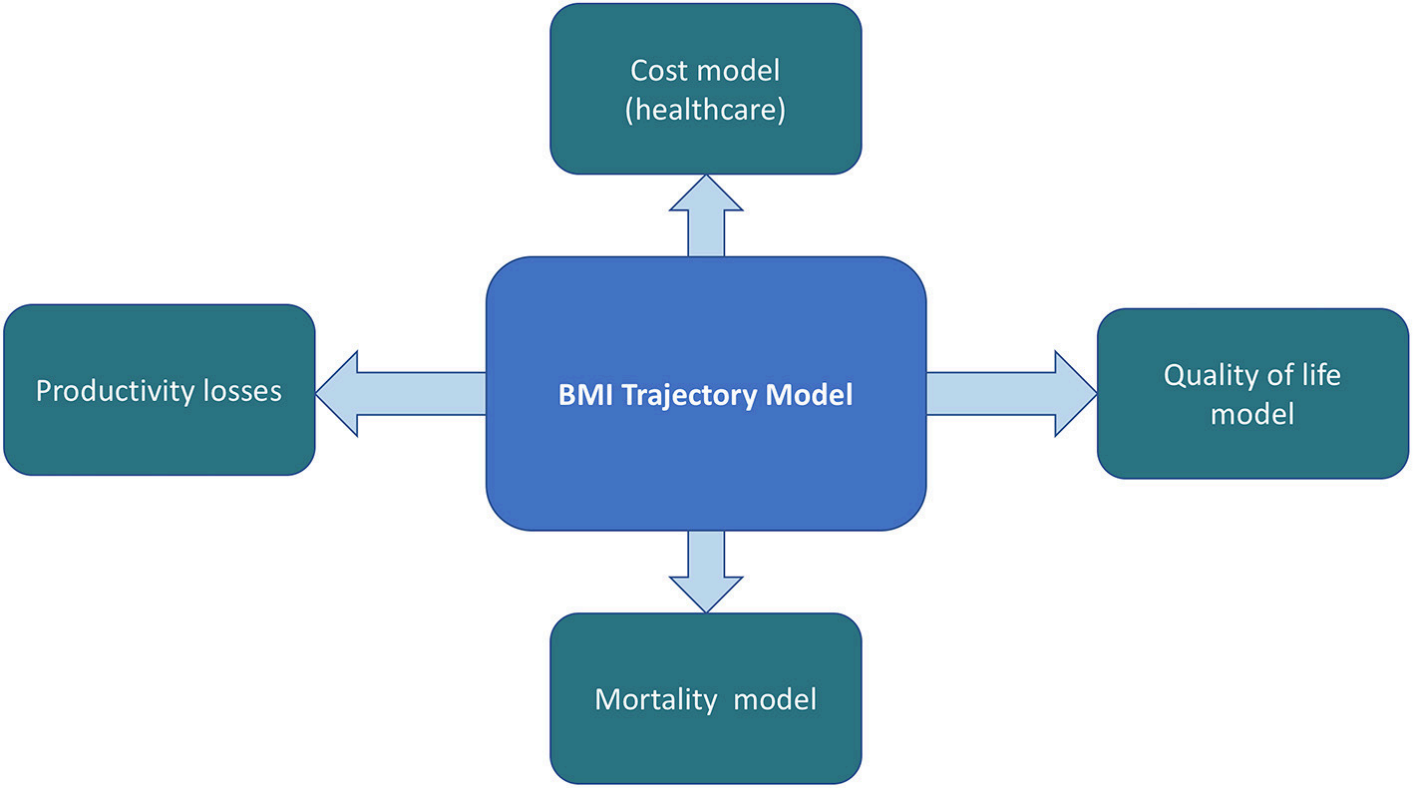
The EPOCH model structure.

The EPOCH model consists of five linked quantitative models for estimating HRQoL outcomes, direct healthcare costs, and productivity costs in relation to child BMI status at different ages. The overarching model structure is shown in Figure 1.

At the core of our proposed model framework is an epidemiological model that predicts BMI trajectories through annual gain in weight (BMI) based on child age, sex, socioeconomic position, and current weight status. The EPOCH model takes the approach, that change in BMI is directly associated with costs and effects, rather than simulating chronic disease events as these will generally not become apparent until adulthood (22). The BMI trajectory model and quality of life sub-models have been completed and are described below; other sub-models for projection of direct and indirect healthcare costs will be successively added. The model is programmed in STATA v14 (37).

### Data Sources Informing the Model

We have used published data from systematic reviews and meta-analyses and new analysis of existing population datasets to develop models to predict BMI and quality of life trajectories from early childhood to adolescence. Healthcare cost trajectories, will use high levels of evidence from systematic reviews and linked data studies where available.

The BMI trajectory model, (described in more detail below) is based on data from the population-representative Australian study, the Longitudinal Study of Australian Children (LSAC) (38), one of the largest longitudinal studies of child development in the world. The study follows two cohorts of children—the infant cohort and child cohort at 2-year intervals from 2004 (Wave 1) until 2014 (Wave 6) being the latest round of data collection. The sampling design is a 2-stage clustered survey design with geographic stratification (39). Data were collected at the child's home through face-to-face interviews, and BMI and BMI z-scores were calculated from measured height and weight, using a portable stadiometer and digital bathroom scales. Mortality is modeled through use of Australian Life Tables (40), using the age and sex specific annual mortality rates which are provided in age intervals of 1 year.

The quality of life sub-model is based on a systematic review of the association between weight status and utility-based quality of life in children (9). This enables calculation of quality adjusted life years (QALYs) by age and sex. Direct health care costs in relation to child BMI will be modeled from published data, for example on the association between direct healthcare costs and early child obesity in Australia (31, 32, 41), and also drawing on studies from overseas (33, 42–44). The valuation of indirect healthcare costs in relation to childhood obesity is a relatively unstudied area, yet important to include for the societal perspective as sickness in children will almost certainly result in a loss of productivity or absenteeism for one or both parents or carers (42). To date no studies have included indirect costs in childhood models but The Australia Household, Income and Labor Dynamics survey and published data from overseas will be investigated as sources of data to model these indirect costs.

### Running the Model

The model has the flexibility to be initialized and run with starting populations of children of any age from 4 years upwards that include individual-level data on measured body mass index (BMI). The different input data sources could be nationally-representative samples of children, for example, LSAC (see below) or National Health Surveys (1), or individual level data from randomized control trials. The model projects BMI trajectories for each individual in the data set, using discrete time with annual cycles. This means it predicts BMI year by year and without further input from the source dataset. Annual healthcare costs, which may be direct and indirect (depending on the perspective taken) in relation to weight status and age will be computed in each annual time-step. Similarly, quality adjusted life years (QALYs), are based on utility weights by age and weight status (9). Model outputs will include, BMI, quality of life and cost trajectories. It is also possible to determine prevalence of weight status groups by age (underweight, healthy weight, overweight, obesity, and severe obesity) according to World Health Organization (WHO) BMI-for-age cut points (45). When running the model with survey data, outputs of interest such as mean BMI or prevalence of obesity can be inferred at a population level, by using survey estimation techniques (svy command in STATA (37) and the survey weights attributed to each individual in the simulation. These are established methods that can combine individual-level simulation with survey estimation techniques and have been used to model obesity progression in a nationally-representative adult population (46). This offers the possibility of joining the two models in the future such that modeling from childhood through to the transition to early adulthood (47) would be possible.

### Using the Model for Economic Evaluation

**Figure 2:**
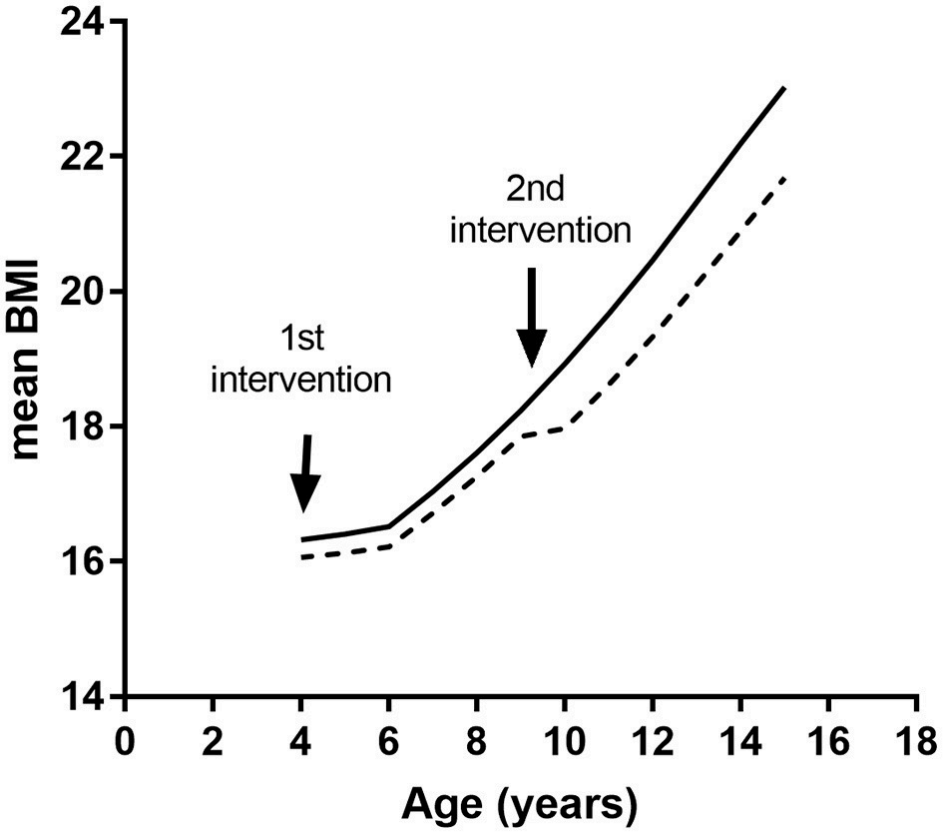
Simulated BMI trajectories resulting from two interventions. Solid line, control trajectory; dashed line, trajectory with hypothetical interventions at ages 4 and 9 years.

Modeled economic evaluations will be able to be undertaken from a health care payer perspective (including direct healthcare costs) and from a societal perspective (including productivity losses). We will use standard economic methods and a micro-costing approach to determine the components of an intervention, their unit costs, and mean total costs per participant to deliver a particular intervention. The EPOCH model will predict downstream costs and health outcomes in relation to simulated BMI. Typically, this will involve running the model with and without intervention effects overlaid and then determining incremental costs and outcomes for an intervention compared with usual care, so that incremental cost-effectiveness ratios may be calculated. We will review the literature for evidence of effectiveness of interventions in early childhood, based on systematic reviews or other published literature. The effects of an intervention, in terms of reduction in expected age-specific weight gain—or weight loss—will be overlaid onto the predicted BMI trajectory, so expected BMI change per annum is adjusted for the duration that effectiveness is sustained. Interventions can be evaluated singly or in combination by successively applying BMI reductions representing intervention effects at the appropriate age. An example of the results of applying an intervention effect from an early childhood intervention followed by an intervention in primary school is shown in Figure 2.

Incremental cost-effectiveness ratios (ICERs) can either be determined for a discrete group of study children, e.g., a small target population such as a RCT sample, or at a population level. In the case of the latter, this will require knowledge of the size of the target population, the cost of delivering the intervention at scale, the size of intervention effects when implemented, and persistence of the intervention effect. The most conservative assumption is that changes in BMI will persist only for the duration of the intervention, but other scenarios may also be investigated. Sensitivity analysis will be able to investigate the impact of these different assumptions regarding sustainability.

ICERs will be calculated in terms of cost per QALY saved, cost per unit BMI avoided or cost per BMI z-score unit avoided, at a future pre-specified age. Discounting of costs and effects beyond 1 year, will be performed. Appropriate sensitivity analysis will be performed, by changing effect sizes by their upper and lower confidence intervals.

## Development and Validation of the BMI Trajectory Model

In this section we describe the development of the BMI trajectory model and internal validation. Validation is an important step in model building as it ensures outputs are consistent with available data, and thus gives confidence in predictions beyond available data. We believe it is particularly important to validate the epidemiological predictions of the model before extending its use to health economic outcomes, as future healthcare costs and QALYs are dependent on predictions of BMI.

Research has shown that there are differences in BMI trajectories among countries. For example, in a study of seven European countries, Börnhorst et al. (48) found large between-country differences when they estimated BMI growth curves for children aged 0–12 years old. Hence it is important to use Australian-specific data in our modeling. Equations for weight (BMI) gain underpinning our model were derived from the LSAC (Table 1).

**Table 1:** LSAC respondents by wave of data collection.

	**Wave 1**	**Wave 2**	**Wave 3**	**Wave 4**	**Wave 5**	**Wave 6**
	**2004**	**2006**	**2008**	**2010**	**2012**	**2014**
**INFANT COHORT**						
Participants (#)	5,107	4,514	4,311	4,171	3,988	3,562
Age range (years)	0–1	2–3	4–5	6–7	8–9	10–11
Mean BMI	.	16.9	16.4	16.6	17.7	19.0
Overweight (%)	.	29.7	26.2	19.8	20.9	22.8
Obese (%)	.	13.6	9.4	10.6	12.8	12.9
**CHILD COHORT**						
Participants (#)	4,934	4,423	4,289	4,018	3,802	3,268
Age range (years)	4–5	6–7	8–9	10–11	12–13	14–15
Mean BMI	16.3	16.6	17.7	19.2	20.6	22.2
Overweight (%)	23.8	19.9	21.9	22.9	21.9	20.9
Obese (%)	9.1	9.6	13.4	15.0	12.4	11.7

### Equations for Annual BMI Change

We extracted information on age, sex, BMI, and socioeconomic position of children from both cohorts. Observations that had implausible BMI z-scores (>5 or <-5) were excluded (<0.1% of all observations). Change in BMI between waves was determined for each individual child, then stratified by change in BMI for individual years of age. As age-specific BMI and BMI change per annum were not significantly different between the infant and child cohorts (Wald test *p* > 0.05), and to maximize our sample size, we combined the data from both cohorts for the analysis. We derived multivariable equations to predict annual change in BMI based on individual child characteristics of age, sex, and current BMI status. Two equations were derived for boys, and 3 equations for girls covering the age range from 4 years to 15 years. As a first step we simulated and validated the BMI trajectories, without stratification by socio-economic position.

### Internal Validation of BMI Trajectory Model

**Figure 3:**
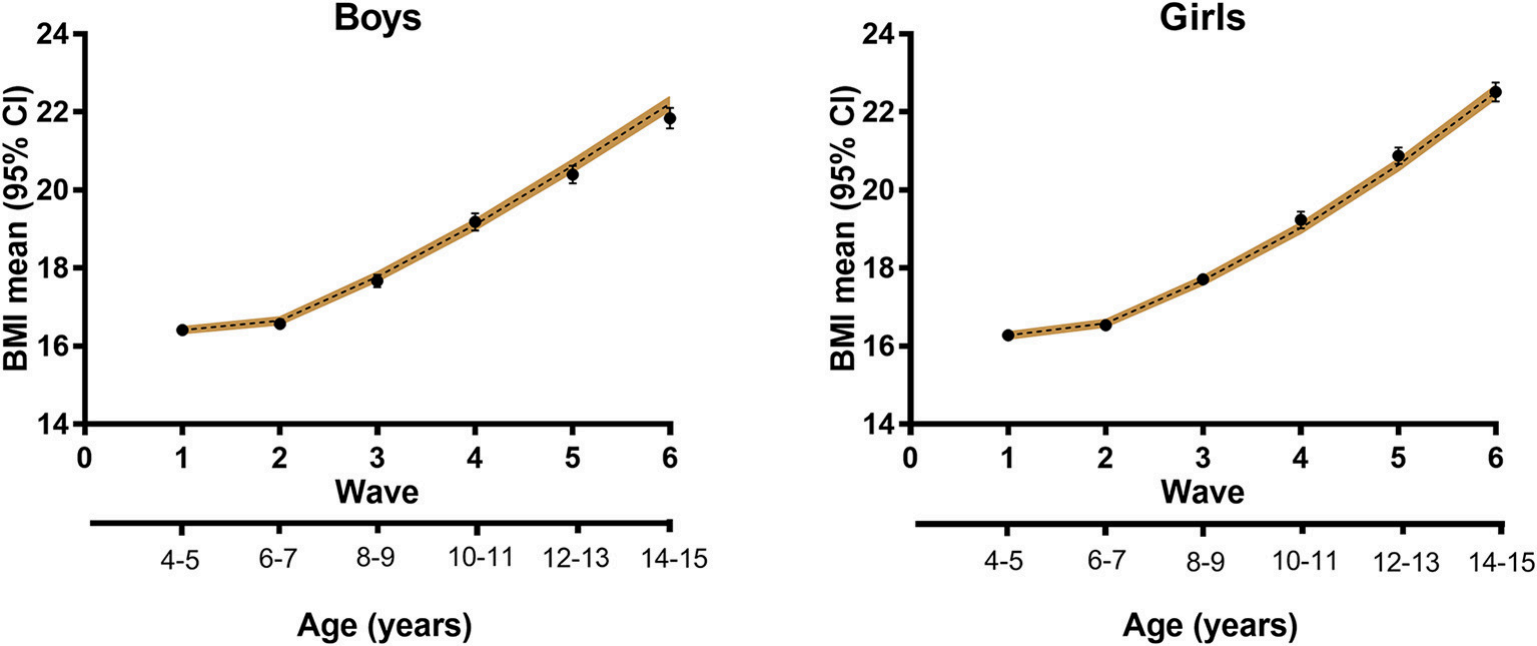
Modeled BMI trajectories for boys and girls. Black circles, data from LSAC; dashed line, modeled trajectory with 95% confidence interval (shaded).

The input population was individual-level data on children aged 4/5 years from the child cohort of LSAC. The model was initialized with a dataset of 4,983 participants from Wave 1 (Table 1), representing a population of over 250,000 children). We simulated BMI trajectories from age 4/5 years to 14/15 years, and compared the predictions to the data from five more waves of the child cohort of the LSAC (Figure 3). Similarly, simulated healthy weight, overweight and obesity prevalence based on WHO growth standards (45) were compared with observed data. Prevalence data (both simulated and from the surveys) were determined using survey estimation (svy command in Stata).

## Results

The modeled results showed good internal validation in terms of mean BMI trajectories, the changing BMI distribution over time and predicted trends in obesity prevalence. For example, starting with mean BMI at age 4/5 years of 16.4 and 16.3 kg/m^2^ for boys and girls, respectively, the EPOCH model predicted that mean BMI 10 years later would be 22.2 and 22.5 kg/m^2^, within the 95% confidence interval of the LSAC data of 21.8 kg/m^2^ (95% CI 21.6–22.1) for boys and 22.5 kg/m^2^ (95% CI 22.3–22.8) for girls (Figure 3).

**Figure 4:**
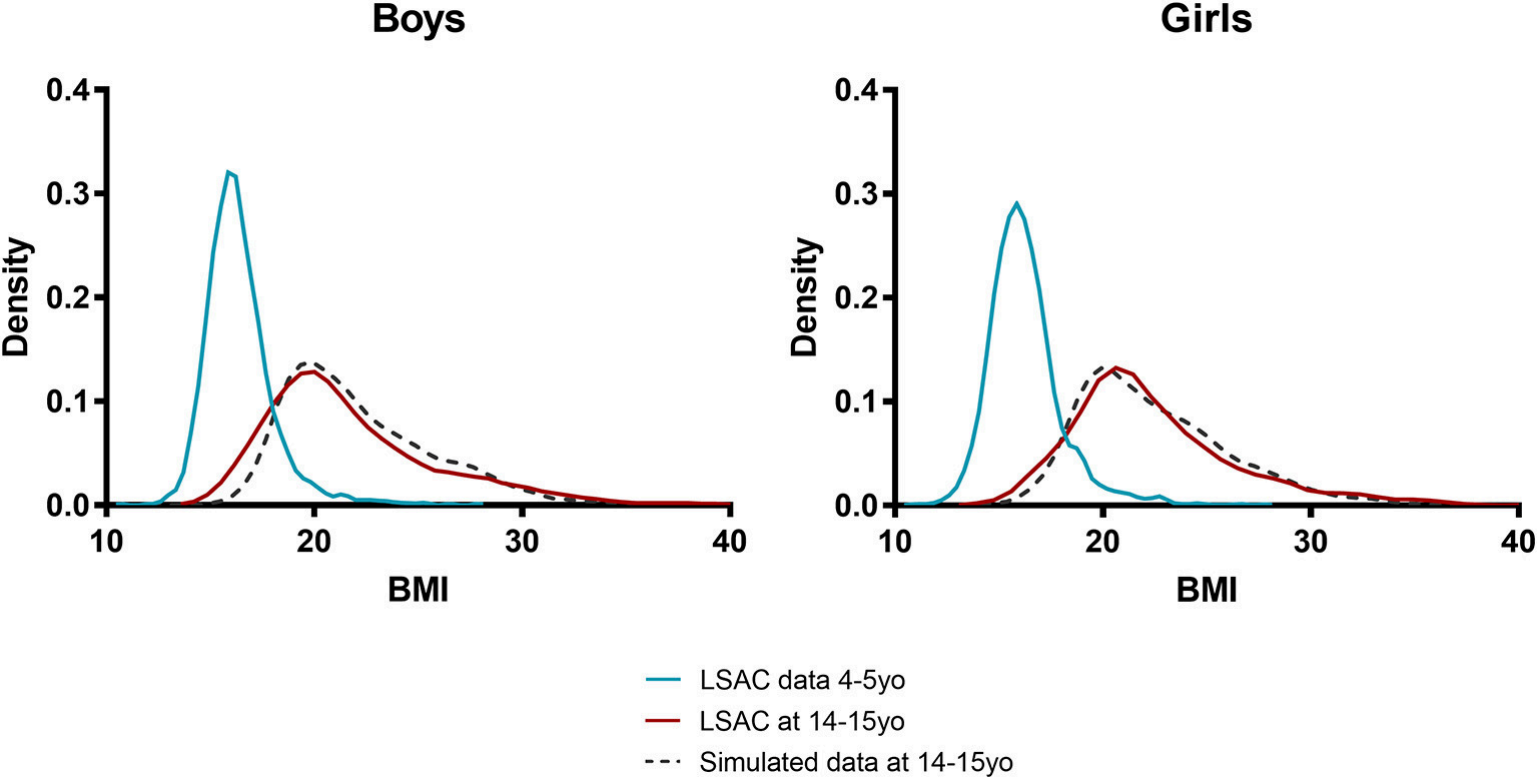
Simulated and actual BMI distribution for boys and girls. Blue line, input population distribution of 4 and 5 year olds; red line, actual distribution at age 14/15 years; dashed black line, simulated distribution at age 14/15 years.

Similarly, the modeled population distribution of BMI in adolescence corresponded to the actual population distribution determined from LSAC. From a very tight distribution in early childhood the model was able to simulate the increasing right skew of this distribution in adolescence (Figure 4).

The predicted trends in obesity prevalence during childhood also corresponded well to survey data. For example, the model projected the prevalence of obesity to increase over a 10-year period from 10.2 to 13.5% for boys and from 8.4 to 9.0% for girls. This is consistent with the observed prevalence and 95% confidence intervals in LSAC of 13.2% (95% CI 11.2–15.5) and 10.0% (95% CI 8.3–12.0) for boys and girls, respectively. It is worth noting that due to the very low mortality rate in Australian children, the modeled population at age 14/15 years is very similar in number to the base, starting population at 4/5 years. Yet, attrition in the LSAC survey population meant that Wave 6 of the child cohort had almost 30% loss to follow-up, and hence the population available for measurement, may have been biased.

## Discussion

In this paper, we have described the rationale and conceptual structure of the EPOCH model, with which we aim to generate new evidence to inform policy on the most effective and cost-effective ways to reduce childhood obesity. We have validated the epidemiological predictions of the model from early childhood until adolescence. The model was accurate in its predictions of average BMI and obesity trajectories, and was able to predict changing BMI distribution from age 4 years through to age 15 years.

To our knowledge this is the first micro-simulation model to predict BMI and obesity trajectories over childhood and adolescence. Micro-simulation, whilst more demanding in terms of software/coding skills (49), offers the advantage of capturing full heterogeneity of the modeled population and accounting for individual-level variation in costs and outcomes. Whilst the data requirements of micro-simulation are higher than other modeling methodologies, we have access to 10 years of very rich longitudinal data from the LSAC with repeated measures of individual child BMI. Previous studies have modeled BMI and growth trajectories (50, 51), but these are usually based on statistical modeling, or latent class analysis (52) and do not offer the same level of flexibility to investigate the impact of single or multiple interventions at different ages nor to predict health economic outcomes.

The strengths of our study include the novel use of micro-simulation modeling that accounts for heterogeneity in the modeled populations. The EPOCH model is not restricted to predicting three weight status categories as some models are (24, 29) but can model the full population distribution of BMI including the upper range of BMI. Whilst severe obesity among children is currently at low prevalence, (around 2%) it has severe impacts on health and well-being for the individual. The EPOCH model will have the flexibility to investigate the impact of targeted interventions in these groups. Another novel aspect of the EPOCH model is the use of the survey weights provided in LSAC, which makes the predictions of our model generalizable at the population level.

Another major strength of the study is the validation of the epidemiological predictions of the model prior to incorporating health economic components. Whilst there is debate in the literature on the need to validate models before using them (22, 53), we believe this increases confidence in using the model for economic outcomes which will be modeled via BMI and/or weight status. It also demonstrates the potential of using this model to predict likely future weight status beyond early childhood, which may be useful for planning and management of obesity. Thus, beyond its use for economic evaluation, the model could be used for planning, and has the potential to offer new perspectives on strategies for reducing obesity development during childhood and adolescence. A final strength is that we have followed established guidelines for choosing a conceptual structure of the model (21, 54) and reporting the model (55).

A limitation of the study is that the BMI trajectory component of the EPOCH model has presently only been validated up to 15 years of age. This limitation is due to the present availability of the LSAC data waves. However, the model has a flexible structure that will allow for incorporation of additional weight gain equations for children 15 years and older, as new waves of data from LSAC become available. Similarly, as the LSAC contains information on children's socio-economic position, future work will include updating model equations and parameters to include a measure of socio-economic position.

This detailed epidemiological model, which is the basis of a health economic model will fill a gap in the literature, because existing models do not usually taken account of healthcare and other cost savings incurred during the childhood years, as a result of treatment or prevention activities. As such this work will complement findings from other modeling efforts within Australia, for example the “ACE-Obesity Policy model” (56) which tracks cost and benefits accrued over the longer term.

Future use of the EPOCH model for economic evaluation will entail completion of the, healthcare costs and productivity cost sub-models. It will also involve systematic or scoping reviews on effect size and effect decay among interventions in the under 5 age group. The EPOCH model will be able to compare obesity interventions in early childhood and later childhood, by predicting BMI trajectories starting from any age and comparing costs and outcomes at the same future point in time. Modeling will enable us to compare interventions that vary in their target age, their intensity and duration and to elucidate the best combination of interventions from a health payer or a societal perspective. It will have the capacity to evaluate interventions singly or in combination and will be able to identify the potential cost-effectiveness of a range of interventions before they are implemented at full-scale. Ultimately the EPOCH model will assist policy makers in identifying: when is it best to intervene in childhood; what are the most cost-effective approaches and which population groups will benefit most from interventions.

## Ethics Statement

Ethics approval for the study has been granted by the University of Sydney Human Research Ethics Committee (2018/726).

## Author Contributions

AH conceived the study. AH and ET analyzed the data and conducted the modeling. TL, AH, and ET wrote the software code. AH wrote the first draft of the manuscript. VB, MM, TL, and LB critically revised the manuscript. All authors contributed to manuscript revision, read, and approved the submitted version.

### Conflict of Interest Statement

The authors declare that the research was conducted in the absence of any commercial or financial relationships that could be construed as a potential conflict of interest.

## References

[1] Australian Bureau of Statistics National Health Survey: First Results, 2014-15. Canberra, ACT: ABS (2015).

[2] NCD Risk Factor Collaboration Worldwide trends in body-mass index, underweight, overweight, and obesity from 1975 to 2016: a pooled analysis of 2416 population-based measurement studies in 128.9 million children, adolescents, and adults. Lancet. (2017) 390:2627–42. 10.1016/S0140-6736(17)32129-3 29029897 PMC5735219

[3] World Health Organization Report of the Commission on Ending Childhood Obesity. Geneva: WHO (2016).

[4] Waters E de Silva-Sanigorski A Hall BJ Brown T Campbell KJ Gao Y Interventions for preventing obesity in children. Cochrane Database Syst Rev. (2011) 12:CD001871 10.1002/14651858.CD001871.pub3 22161367

[5] John J Wolfenstetter SB Wenig CM. An economic perspective on childhood obesity: recent findings on cost of illness and cost effectiveness of interventions. Nutrition. (2012) 28:829–39. 10.1016/j.nut.2011.11.016 22452837

[6] Döring N Mayer S Rasmussen F Sonntag D. Economic evaluation of obesity prevention in early childhood: methods, limitations and recommendations. Int J Environ Res Public Health. (2016) 13:911. 10.3390/ijerph13090911 27649218 PMC5036744

[7] Hesketh KD Campbell KJ. Interventions to prevent obesity in 0-5 year olds: an updated systematic review of the literature. Obesity. (2010) 18:S27–35. 10.1038/oby.2009.429 20107458

[8] Bond M Wyatt K Lloyd J Welch K Taylor R. Systematic review of the effectiveness and cost-effectiveness of weight management schemes for the under-fives: a short report. Health Technol Assess. (2009) 13:1–75 10.3310/hta13610 20015425

[9] Brown V Tan EJ Hayes AJ Petrou S Moodie ML. Utility values for childhood obesity interventions: a systematic review and meta-analysis of the evidence for use in economic evaluation. Obes Rev. (2018) 19:905–16. 10.11111/obr.12672. 29356315

[10] Burke V. Obesity in childhood and cardiovascular risk. Clin Exp Pharmacol Physiol. (2006) 33:831–7. 10.1111/j.1440-1681.2006.04449.x 16922816

[11] Hao G Wang X Treiber FA Harshfield G Kapuku G Su S. Body mass index trajectories in childhood is predictive of cardiovascular risk: results from the 23-year longitudinal georgia stress and heart study. Int J Obes. (2017) 42:923–5. 10.1038/ijo.2017.244 28978977 PMC5886821

[12] Huang RC De Klerk NH Smith A Kendall GE Landau LI Mori TA. Lifecourse childhood adiposity trajectories associated with adolescent insulin resistance. Diabetes Care. (2011) 34:1019–25. 10.2337/dc10-1809 21378216 PMC3064016

[13] Reilly J Methven E. Health consequences of obesity. Arch Dis Child. (2003) 88:748–52. 10.1136/adc.88.9.748 12937090 PMC1719633

[14] Whitaker RC Pepe MS Wright JA Seidel KD Dietz WH. Early adiposity rebound and the risk of adult obesity. Pediatrics. (1998) 101:e5. 10.1542/peds.101.3.e5 9481024

[15] Ong K Loos R. Rapid infancy weight gain and subsequent obesity: systematic reviews and hopeful suggestions. Acta Paediatr Int J Paediatr. (2006) 95:904–8. 10.1080/08035250600719754 16882560

[16] Stettler N Iotova V. Early growth patterns and long-term obesity risk. Curr Opin Clin Nutr Metab Care. (2010) 13:294–9. 10.1097/MCO.0b013e328337d7b9 20179588

[17] Reilly JJ Kelly J. Long-term impact of overweight and obesity in childhood and adolescence on morbidity and premature mortality in adulthood: systematic review. Int J Obes. (2011) 35:891–8. 10.1038/ijo.2010.222 20975725

[18] Davies R Roderick P Raftery J The evaluation of disease prevention and treatment using simulation models. Eur J Oper Res. (2003) 150:53–66. 10.1016/S0377-2217(02)00783-X

[19] Levy DT Mabry PL Wang YC Gortmaker S Huang TTK Marsh T. Simulation models of obesity: a review of the literature and implications for research and policy. Obes Rev. (2011) 12:378–94. 10.1111/j.1467-789X.2010.00804.x 20973910 PMC4495349

[20] Gortmaker SL Swinburn BA Levy D Carter R Mabry PL Finegood DT. Changing the future of obesity: science, policy and action. Lancet. (2011) 378:838–47. 10.1016/S0140-6736(11)60815-5 21872752 PMC3417037

[21] Roberts M Russell LB Paltiel AD Chambers M McEwan P Krahn M. Conceptualizing a model: a report of the ISPOR-SMDM modeling good research practices task force-2. Med Decis Mak. (2012) 32:678–89. 10.1177/0272989X12454941 22990083

[22] Schwander B Hiligsmann M Nuijten M Evers S. Systematic review and overview of health economic evaluation models in obesity prevention and therapy. Exp Rev Pharmacoecon Outcomes Res. (2016) 16:561–70. 10.1080/14737167.2016.1230497 27570095

[23] Hollinghurst S Hunt LP Banks J Sharp DJ Shield JP. Cost and effectiveness of treatment options for childhood obesity. Pediatr Obes. (2014) 9:26–34. 10.1111/j.2047-6310.2013.00150.x 23505002

[24] Pil L Putman K Cardon G De Bourdeaudhuij I Manios Y Androutsos O. Establishing a method to estimate the cost-effectiveness of a kindergarten-based, family-involved intervention to prevent obesity in early childhood. The ToyBox-study. Obes Rev. (2014) 15:81–89. 10.1111/obr.12179 25047383

[25] Carter R Vos T Moodie M Haby M Magnus A Mihalopoulos C. Priority setting in health: origins, description and application of the Australian Assessing Cost–effectiveness initiative. Exp Rev Pharmacoecon Outcomes Res. (2008) 8:593–617. 10.1586/14737167.8.6.593 20528370

[26] Haby MM Vos T Carter R Moodie M Markwick A Magnus A. A new approach to assessing the health benefit from obesity interventions in children and adolescents: the assessing cost-effectiveness in obesity project. Int J Obes. (2006) 30:1463–75. 10.1038/sj.ijo.0803469 17003807

[27] Moodie M Haby M Galvin L Swinburn B Carter R. Cost-effectiveness of active transport for primary school children - Walking School Bus program. Int J Behav Nutr Phys Act. (2009) 6:63. 10.1186/1479-5868-6-63 19747402 PMC2758827

[28] Cobiac LJ Vos T Barendregt JJ. Cost-effectiveness of interventions to promote physical activity: a modelling study. PLoS Med. (2009) 6:e1000110. 10.1371/journal.pmed.1000110 19597537 PMC2700960

[29] Hollingworth W Hawkins J Lawlor DA Brown M Marsh T Kipping RR Economic evaluation of lifestyle interventions to treat overweight or obesity in children. Int J Obes. (2012) 36:559–66. 10.1038/ijo.2011.272 22249222

[30] Singh AS Mulder C Twisk JWR Van Mechelen W Chinapaw MJM. Tracking of childhood overweight into adulthood: a systematic review of the literature. Obes Rev. (2008) 9:474–88. 10.1111/j.1467-789X.2008.00475.x 18331423

[31] Au N. The health care cost implications of overweight and obesity during childhood. Health Serv Res. (2012) 47:655–76. 10.1111/j.1475-6773.2011.01326.x 22092082 PMC3419882

[32] Hayes A Chevalier A D'Souza M Baur LA Wen LM Simpson JM. Early childhood obesity: association with healthcare expenditure in Australia. Obesity. (2016) 24:1752–8. 10.1002/oby.21544 27380909

[33] Trasande L Chatterjee S. The impact of obesity on health service utilization and costs in childhood. Obesity. (2009) 17:1749–54. 10.1038/oby.2009.67 19300433

[34] Kuhle S Fung C Veugelers PJ. Medication use in normal weight and overweight children in a nationally representative sample of Canadian children. Arch Dis Child. (2012) 97:842–7. 10.1136/archdischild-2011-301195 22833408

[35] Grutters JPC Sculpher M Briggs AH Severens JL Candel MJ Stahl JE. Acknowledging patient heterogeneity in economic evaluation: a systematic literature review. Pharmacoeconomics. (2013) 31:111–123. 10.1007/s40273-012-0015-4 23329430

[36] Hardy LL Mihrshahi S Gale J Drayton BA Bauman A Mitchell J. 30-year trends in overweight, obesity and waist-to-height ratio by socioeconomic status in Australian children, 1985 to 2015. Int J Obes. (2017) 41:76–82. 10.1038/ijo.2016.204 27847388 PMC5220161

[37] StataCorp Stata Statistic Software: Release 14.2. College Station. Texas, TX: StataCorp LP (2016).

[38] Edwards B Growing up in Australia: the longitudinal study of Australian children: the first decade of life. Fam Matt. (2012) 91:7–17.

[39] Soloff C Lawrence D Johnstone R Sample Design (LSAC Technical Paper, No. 1). Melbourne, VIC: Australian Institute of Family Studies (2005).

[40] Australian Bureau of Statistics Life Tables, States, Territories and Australia, 2015-17 (Cat. No. 3302.0.55.001). Canberra, ACT: Australian Bureau of Statistics (2018).

[41] Clifford SA Gold L Mensah FK Jansen PW Lucas N Nicholson JM. Health-care costs of underweight, overweight and obesity: Australian population-based study. J Paediatr Child Health. (2015) 51:1199–206. 10.1111/jpc.12932 26059311

[42] Hamilton D Dee A Perry IJ. The lifetime costs of overweight and obesity in childhood and adolescence: a systematic review. Obes Rev. (2017) 19:452–63. 10.1111/obr.12649. 29271111

[43] Finkelstein EA Graham WCK Malhotra R. Lifetime direct medical costs of childhood obesity. Pediatrics. (2014) 133:854–62. 10.1542/peds.2014-0063 24709935

[44] Sonntag D Ali S Lehnert T Konnopka A Riedel-Heller S König HH. Estimating the lifetime cost of childhood obesity in Germany: results of a Markov model. Pediatr Obes. (2015) 10:416–22. 10.1111/ijpo.278 25612250

[45] World Health Organization Growth Reference Data for 5-19 Years 2006. Available online at: http://www.who.int/childgrowth/en/ (Accessed December 1, 2015).

[46] Hayes AJ Lung TWC Bauman A Howard K. Modelling obesity trends in Australia: unravelling the past and predicting the future. Int J Obes. (2017) 41:178–85. 10.1038/ijo.2016.165 27671035

[47] Ward ZJ Long MW Resch SC Giles CM Cradock AL Gortmaker SL. Simulation of growth trajectories of childhood obesity into adulthood. N Engl J Med. (2017) 377:2145–53. 10.1056/NEJMoa1703860 29171811 PMC9036858

[48] Börnhorst C Siani A Russo P Kourides Y Sion I Molnár D. Early life factors and inter-country heterogeneity in BMI growth trajectories of European children: the IDEFICS study. PLoS ONE. (2016) 11:e0149268. 10.1371/journal.pone.0149268 26901773 PMC4762899

[49] Spielauer M What is social science microsimulation? Soc Sci Comput Rev. (2011) 29:9–20. 10.1177/0894439310370085

[50] Giles LC Whitrow MJ Davies MJ Davies CE Rumbold AR Moore VM. Growth trajectories in early childhood, their relationship with antenatal and postnatal factors, and development of obesity by age 9 years: results from an Australian birth cohort study. Int J Obes. (2015) 39:1049–56. 10.1038/ijo.2015.42 26008137

[51] Magee CA Caputi P Iverson DC. Identification of distinct body mass index trajectories in Australian children. Pediatr Obes. (2013) 8:189–98. 10.1111/j.2047-6310.2012.00112.x 23143781

[52] Liu JX Liu JH Frongillo EA Boghossian NS Cai B Hazlett LJ. Body mass index trajectories during infancy and pediatric obesity at 6 years. Ann Epidemiol. (2017) 27:708–15. 10.1016/j.annepidem.2017.10.008 29173577

[53] Caro JJ Briggs AH Siebert U Kuntz KM. Modeling good research practices - overview: a report of the ISPOR-SMDM modeling good research practices task force-1. Value Heal. (2012) 15:796–803. 10.1016/j.jval.2012.06.012 22999128

[54] Briggs ADM Wolstenholme J Blakely T Scarborough P. Choosing an epidemiological model structure for the economic evaluation of non-communicable disease public health interventions. Popul Health Metr. (2016) 14:1–12. 10.1186/s12963-016-0085-1 27152092 PMC4857239

[55] Weinstein MC O'Brien B Hornberger J Jackson J Johannesson M McCabe C. Principles of good practice for decision analytic modeling in health-care evaluation: report of the ISPOR task force on good research practices–modeling studies. Value Heal. (2003) 6:9–17. 10.1046/j.1524-4733.2003.00234.x 12535234

[56] Ananthapavan J Sacks G Brown V Moodie M Nguyen P Barendregt J Assessing Cost-Effectiveness of Obesity Prevention Policies in Australia 2018 (ACE-Obesity Policy). Melbourne, VIC: Deakin University (2018).

